# Burn Model for In Vivo Investigations of Microcirculatory Changes

**Published:** 2009-04-03

**Authors:** Ole Goertz, Julian Vogelpohl, Birger Jettkant, Adrien Daigeler, Hans Ulrich Steinau, Lars Steinstraesser, Stefan Langer

**Affiliations:** ^a^ Department of Plastic and Hand Surgery, Burn Center; ^b^ Department of Surgery, BG-University Hospital Bergmannsheil, Ruhr University Bochum, Buerkle-de-la-Camp-Platz 1, Bochum 44789, Germany

## Abstract

**Objective:** The treatment of burns remains a challenge due to the associated high morbidity and mortality. Besides the administration of physiologic saline, local disinfection, and symptomatic medications, no causal therapy is known to reduce the tissue damage and accelerate wound healing. The aim of the study was to develop a reliable burn model that allows for reproducible quantitative in vivo analysis of the microcirculation, angiogenesis, and leukocyte endothelium interaction after burn injury. **Methods:** Experiments were carried out on male hairless mice (*n* = 9). Full-thickness burns were inflicted with a hot air jet without any contact to the tissue (117 ± 2.1°C for 1 second; burn area: 1.3 mm^2^). Intravital fluorescent microscopy, in combination with FITC-dextran as plasma marker, was used to assess microcirculatory standard parameters; leukocytes were stained with rhodamine 6G. Values were obtained before, immediately after, as well as at days 1, 3, 7, and 14 postburn. **Results:** The nonperfused area decreased during the observed period and perfusion was almost completely due to angiogenesis at day 14. No posttraumatic expansion of the nonperfused area after 24 hours could be observed. Leukocyte endothelium interaction showed its maximum 24 hours postburn. The formation of edema occurred immediately postburn and decreased during the following observation time. **Conclusion:** The developed burn model allows a reproducible assessment with significant results of the microcirculation, angiogenesis, and leukocyte endothelium interaction without causing mechanical damage to the tissue; therefore, this model qualifies for the further investigations of interventional drugs to decrease the effects of burn injury.

In 1953, Douglas Jackson published his profound scientific observations of burns and described the 3 different functional zones: the zone of coagulation in the center of the burn wound, the peripheral zone of hyperemia, and the intermediate zone of stasis.^1^ He found this classification by thorough clinical observations and histologic investigations.

Our aim was to detect the intravital pathophysiologic correlate for his results and complete the static and morphologic knowledge about burns by dynamic investigations.

We therefore sought to develop a no-touch burn model that allows repetitive and highly reproducible quantitative in vivo analysis of the microcirculation and leukocyte endothelium interaction after burn injury.

## MATERIALS AND METHODS

### Animals

Hairless mice (SKH-1/hr, *n* = 9, body weight = 18–20 g) were used (Charles River, Sulzfeld, Germany). The animals had access to standard laboratory chow and tap water ad libitum. All experiments were carried out with ethical committee approval and met the standards required by the UK CCCR guidelines. In addition, each procedure was approved by the regional authorities according to German animal care regulations, which comply with the international guidelines of animal care and use in scientific experiments (AZ: 50.8735.1, Nr.: 112/4). After finishing the experiments, the animals were killed under general anesthesia by an overdose of pentobarbital.

### Anesthetizing/preparation

Mice were anesthetized by spontaneous inhalation of isoflurane-N_2_O (FIO_2_ = 0.35, 0.015 L/L isoflurane, Forene, Abbott GmbH, Wiesbaden, Germany), wrapped in a cotton gauze pad to minimize convective heat loss and placed on a heated acryl-glass observation platform to maintain a body temperature of 37°C. Subsequently, 3 microsurgical loops (8/0 Ethilon, Ethicon, Norderstedt, Germany) were pulled through the ear for extension. In addition, physiologic saline between the ear and the platform was used to flatten the ear by adhesive forces.

### Applications

Fluorochromes were administered via the tail veins (tube 29G, Braun, Melsungen, Germany). Fifty microliters of FITC-labeled dextran (1.0%, molecular weight = 150 kDa) served as the plasma marker and 50-µL rhodamine 6G (0.5%) was used for staining leukocytes (both: Sigma Chemicals Co., St Louis, Mo).

### Burn technique

**Figure 1:**
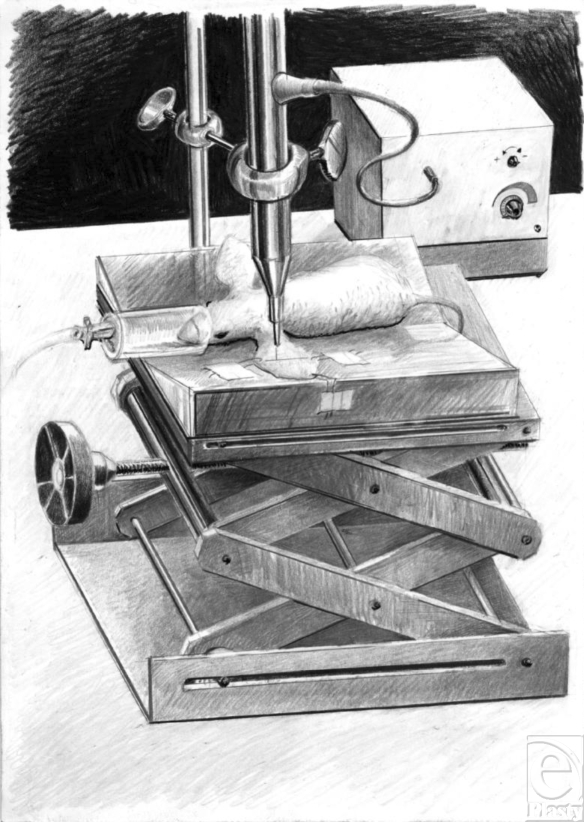
Burn device. Drawing of the experimental setup with the mouse under inhalation anesthesia placed on an acryl-glass platform with the left ear spread out by 3 microsurgical loops. The ear was exposed to an air jet for 1 second at a temperature of 117°C. The distance between the air tube and the surface of the ear is 1 mm.

The full-thickness burn was inflicted on the dorsal side of the ear with a specially designed device, inducing a defined burn injury by a hot air jet without any tissue contact (Fig 1, 117 ± 2.1°C for 1 second; distance between the ear and the air jet = 1 mm; burn area of approximately 1.3 mm^2^).

### Recordings

Before inducing any injury, the prospective burn area was designated and the microcirculatory parameters of the regions of interest were assessed to achieve an intraindividual comparison of the data. For the intravital fluorescence microscopy (Axiotech vario, Carl Zeiss, Oberkochen, Germany), we used a 4-fold objective for the overall view and a 20-fold water-immersion objective (Achroplan 4×/20×, Zeiss), resulting in a total magnification of about 52-fold for the measurement of the nonperfused area (NPA) and 260-fold for microcirculatory measurements, respectively. The *X*-*Y* coordinates of the regions of interest were saved and digital images were obtained to ensure the relocation of the areas throughout the study.

The images were recorded with a charge-coupled video camera (AVT-BC 71, AVT-Horn, Aalen, Germany) and stored digitally. Microscopic observations were performed 5 minutes prior and immediately subsequent to the induction of burn injury as well as during wound healing on days 1, 3, 7, and 14.

### Measurements

**Figure 2:**
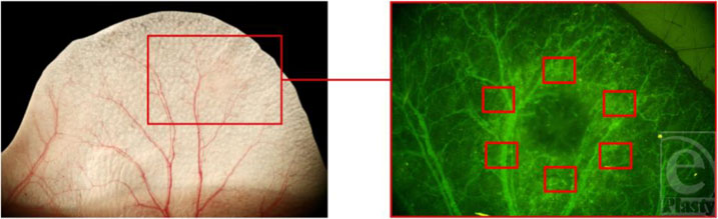
The ear of the hairless mouse in a 10-fold magnification on the left through the operation microscope and in a 52-fold magnification on the right through the fluorescent microscope with the burn area in the middle. The border between the dark area (no perfusion) and the bright FITC-dextran–filled vessels was used for the determination of the nonperfused area. The red boxes (right figure) mark the region of interest.

**Figure 3:**
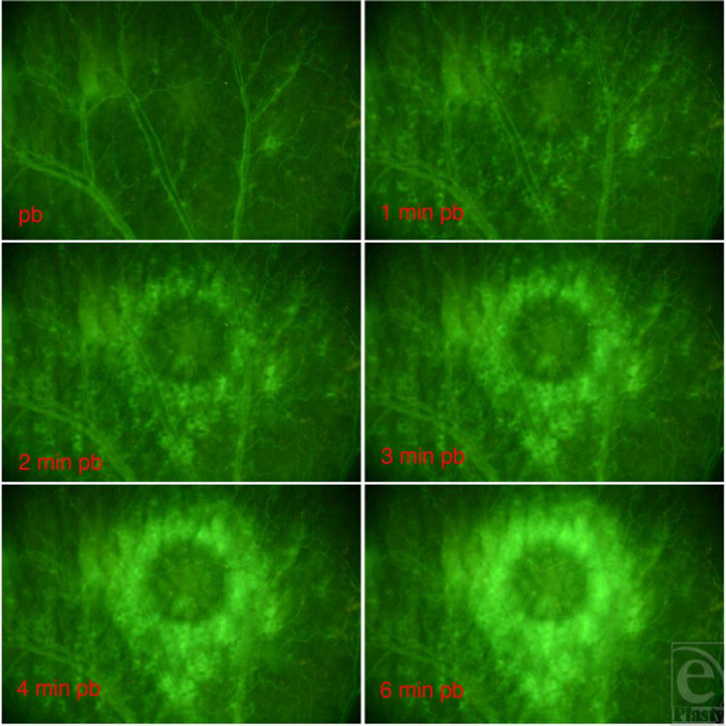
The FITC-dextran–stained ear with the burned area directly postburn in a 52-fold magnification and 1, 2, 3, 4, and 6 minutes postburn. The coagulated, nonperfused tissue appears as a dark circle in the middle of a bright surrounding, where the plasma marker FITC-dextran leaked out of the vessels into the surrounding tissue.

**Figure 4:**
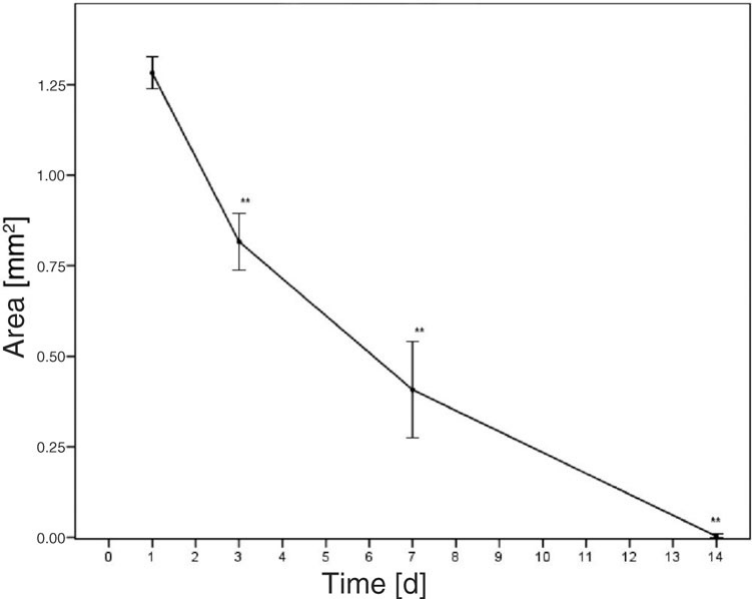
Reduction of the nonperfused area over the observation time due to neovascularization; * * *P* < .01 (*n* = 9).

The NPA was recorded and determined with a digital planimetric mode integrated into the computer-assisted image analysis system CapImage (Figs 2, 3 and 4; Dr Zeintl, Heidelberg, Germany).

Microcirculatory measurements were performed in 6 regions of interest adjacent to the zone of coagulation.^2^ The following parameters were assessed:

1. NPA (*n* = 9) (mm^2^)
2. Functional vessel density (FVD, *n* = 54) (cm/cm^2^)
3. Diameters of arterioles and venules (*n* = 27/*n* = 54) (µm)
4. Midstream red blood cell velocity (RBCV) in venules (*n* = 54) (mm/s)
5. Venular blood flow (*n* = 54) (mL/s)
6. Leakage of plasma marker (*n* = 162) (*I*_*e*_/*I*_*i*_)
7. The number of rolling leukocytes reaching a defined cutoff mark within the venule per minute (*n* = 54) (*n*/min)
8. The number of adherent leukocytes in a defined length of venules per minute (*n* = 54) (*n*/100 µm/per minute)

*Functional vessel density* is defined as the length of perfused vessels per observation area and used as an index for tissue perfusion.^3^ The extravasation of the plasma marker FITC-dextran into the surrounding tissue is a measure of edema formation.^4^ It is expressed as the ratio of the relative fluorescence intensities measured intravasal and extravasal.

### Evaluation

All parameters were analyzed off-line by the computer program CapImage.

### Statistics

The commercially available computer program SPSS, Version 15 (SPSS GmbH, Munich, Germany), was used for the statistical analysis of the data. The mean value, the standard error, and the standard error of the mean of each mouse were calculated for each parameter. To compare the different values with each other, a variance analysis for repeated measurements was used. The mean value of the significant data was compared with the *t* test for paired samples. A *P* < .05 was considered statistically significant.

## RESULTS

The macroscopic evaluation of the burn showed paleness immediately after the injury; in the periphery of the burn, the skin reddened after a few minutes. The intensity of the redness increased during the days that followed and decreased from day 7.

A significant continuous reduction of the NPA over the observation period of 14 days was noted (Figs 2, 3 and 4, *P* < .01, *n* = 9). The angiogenesis was complete 14 days postburn.

The *FVD*, defined as perfused vessels per area (cm/cm^2^) circumjacent to the coagulation zone, decreased immediately postburn to 74.2% of the baseline value (*P* < .01) and increased during the course of observation to a value below the starting point; initial value was not reached during the investigations (Table 1).

The arterial diameter increased significantly by a factor of 1.24 immediately postburn and decreased minimally on each observation time point to reach a value slightly above the baseline on day 14 (Table 1).

The venular diameter increased significantly by 1.13 immediately postburn, but the maximum (with 24% over baseline value) was reached on day 1. The data during the days that followed were lower but significantly over baseline values (Table 1).

The RBCV was measured only in the venules, because the accuracy of measurement was not acceptable in velocities above 0.7 mm/s. The venular RBCV showed a significant increase of 48% immediately postburn and decreased on day 7 to baseline values (Table 1, *P* < .01).

The leakage of the plasma marker FITC-dextran into the extravasal tissue as a parameter for the formation of edema reached the maximum direct posttraumatizing. The leakage decreased on day 1 but remained above baseline values during the entire period of observation (Table 1).

**Figure 5:**
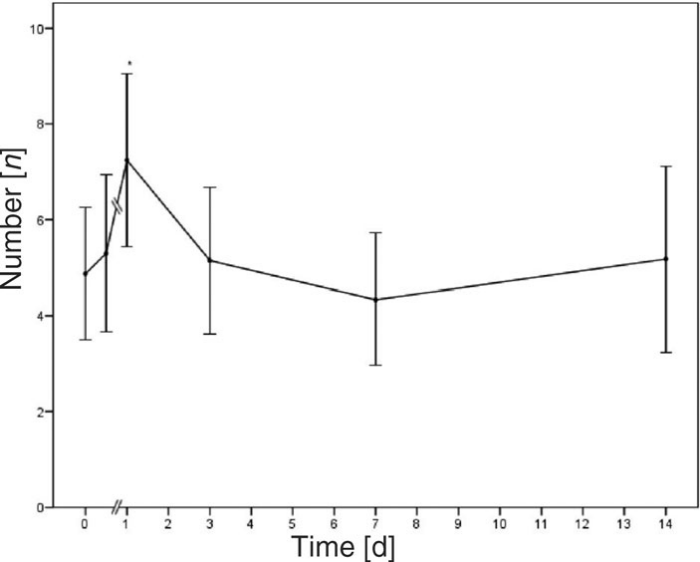
The number of rolling leukocytes within the venules; *P < .05 (*n* = 9, 54 venules).

The number of rolling leukocytes on the endothelium increased immediately subsequent to the burn. The significant maximum was reached 1 day postburn, with an increase of up to 49% over baseline. The data during the days that followed correlated to baseline values (Fig 5).

**Figure 6:**
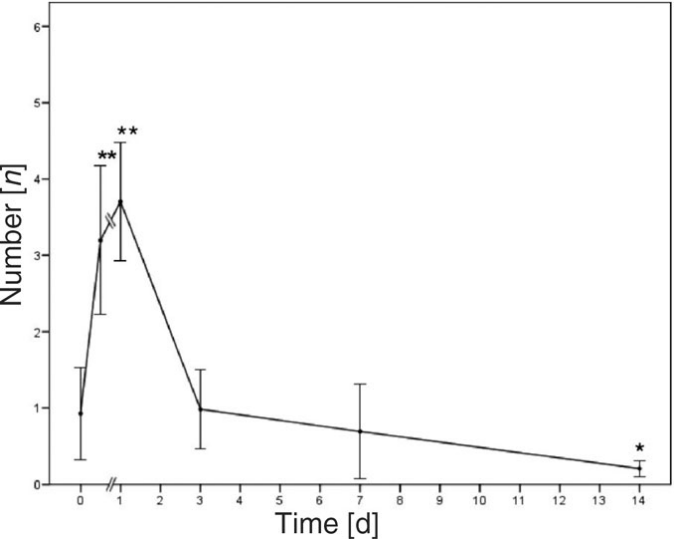
The number of sticking leukocytes at the endothelium per 100-µm venule; *P < .05, * * *P* < .01 (*n* = 9, 54 venules).

The increase in sticking leukocytes was much higher than in the rolling leukocytes. They increased by a factor of 3 direct postburn and by nearly 4 on day 1 postburn. Baseline values were measured on days 3 and 7. On day 14, the number was significantly below baseline values (Fig 6).

## DISCUSSION

The breakdown of skin microcirculation and the increase in leukocytes play an important role in the extension of burns, especially in the zone of stasis and hyperemia, and, consequently, in the prognosis of patients with burn injuries.^5^

The first report on intravital fluorescence microscopy for analyzing perfusion and cell-cell interactions was published in 1839.^6^ Many experimental studies have been carried out since then; most of them were in vitro studies. The first in vivo models in burns were the hamster cheek pouch^7^ and the bat wing in the 1970s of the last century.^8^

In 1980, an intravital scald burn model using the hairless mouse ear was developed,^9^ which allowed to observe correlation between the dynamic changes of the microcirculation and the progressive zones of injury; the analysis of the leukocyte endothelium interaction and repeated investigations during the wound healing were not possible.

Techniques such as orthogonal polarization spectral imaging, fiber optic confocal imaging, ultrasound, and laser Doppler followed.^10^–^13^ The disadvantages of these methods are that they do not allow *direct* visualization of the microcirculation and/or they need *invasive* preparation that itself may cause microcirculatory changes.

In 2005, the first no-touch burn model for in vivo fluorescent microscopy was published.^2^ This model greatly improved the possibility of uninfluenced investigations of the microcirculation but fell short regarding reliability.^2^ It is known that the mechanical trauma caused by the preparation and infliction of the burn plays an important role with regard to the affection of microcirculation and leukocyte behavior.^14^ Therefore, we had to improve the complicated burn technique, which led to insignificant data while maintaining the no-touch technique. Eventually, we were successful with a hot air jet instead of a hot metal tip.

The major parameter for healing, the decrease in size of the NPA due to angiogenesis, showed exactly the same progression like the first model.^2^ The burn size was about 1.3 mm^2^ at day 1 and the perfusion was complete on day 14. The burn in the presented model was inflicted with twice as much heat but only 1% exposure time compared with the first model in the no-touch technique; the microcirculatory results are absolutely comparable (117°C vs 56°C, 1 second vs 100 seconds). The NPA decreased continuously during the observation from day 1 to day 14 and stood in contrast to the often-cited progression of depth, 24 to 48 hours postburn.^15^ The results confirm the clinical and histologic observations of Jackson in 1953, who described no burn expansion after 24 hours.^1^

The FVD serves as an indicator of tissue perfusion.^3^ It stayed significantly below the baseline. The reduction of FVD immediately after the burn may result from arteriole constriction and occlusion by the aggregation of platelets and leukocytes.^9^,^14^,^16^–^19^

The increase in arterial and venular vessel diameters subsequent to a burn has already been shown.^2^,^9^ The upregulation of inducible nitric oxide synthase may cause the vasodilation that sets off an inflammatory cascade that threatens the battered tissue in the zone of stasis and hyperemia.^20^

The venular RBCV showed an increase immediately postburn followed by a reduction during the observation period. In combination with the increased diameter, it indicates a higher blood flow. The increase of diameter is mainly caused by local increase of NO.^21^

The integrity loss of the endothelium immediately postburn with consecutive edema formation was followed by a recovery, but the vessels stayed more permeable than the uninjured tissue. These findings confirmed the data of Boykin and coworkers,^9^ who determined the edema by weighing the harvested tissue. Table 1 shows that the level of the ratio of the relative fluorescence intensities measured inside and outside of the vessels stayed above baseline values for the entire duration of the study. This is not only a result of new edema formation but also a result from the accumulated FITC-dextran in the extravascular tissue due to the repeated injections. The use of intravital fluorescence microscopy for the determination of edema is a decisive advantage in comparison with previous methods of evaluating edema such as weighing, which allows only a single measurement.

The formation of edema is another reason discussed for the burn wound conversion, decreasing intracellular and intravascular fluid resulting in a compromised cellular respiration, and a decreased tissue perfusion.^15^,^22^ The increase in extravasation immediately postburn and the reduction 1 day later support the expectation that the burn conversion primarily occurs during the first 24 hours.

The rolling and sticking of leukocytes to the endothelium take place in uninjured as well as in injured tissue; with much higher numbers in the latter.^23^ The use of fluorescent dyes, such as rhodamine 6G, in combination with light exposure could have phototoxic effects, inducing rolling and sticking of leukocytes.^24^ Therefore, we used only as much dyes as needed, kept the light exposure as short as possible, and conducted the investigations exactly under the same conditions.

The significant increase in leukocytes rolling at and sticking to the inner vessel wall immediately postburn was exceeded 24 hours later by even higher numbers, indicating the peak of the inflammatory process. On day 3, normal values were monitored. The rolling and sticking represent the first 2 steps of the immigration of the leukocytes into the surrounding tissue. The activated neutrophils and xanthine oxidase activity generate oxygen radicals such as hydrogen peroxide and superoxide, which lead to lipid peroxidation and subsequent cell necrosis in burn injuries.^25^

On day 14, the number of sticking leukocytes was significantly lower than the baseline value and approved already published data.^2^ The reduction may be partly due to extensive migration into the tissue, which was observed in histologic sections in HE staining. Prior studies showed that blood coagulation and the recruitment of leukocytes were mostly responsible for the formation of the zone of stasis.^14^,^26^ Contrary to this, we observed only a few adherent leukocytes on the vessel wall, sometimes an accumulation up to 10, but never a complete occlusion of the vessel.

Compared with other models, there are significant advantages: The model allows reliable, direct in vivo investigations of the pathophysiology of microcirculation after burns over the time of healing. The disadvantage lies in the small burn area, which does not allow further interventional investigations, for example, tissue samples for histology.

In summary, the presented burn model using this no-touch technique is reliable and reproducible, leading to significant results. Studies of microcirculation, angiogenesis, and leukocyte endothelium interaction of all burn zones always returning to the same vessel are feasible during healing. The data were underlined by scientific observations published in the past.^2^

The burn model could complete histologic and molecular findings of other models by direct visualization of the effects of drugs, which could help reduce the progression of depth of the burn within the first day and accelerate the healing of burns.

## Acknowledgment

The authors thank Amanda Daigeler, Ottawa, Canada, for editorial assistance.

## Figures and Tables

**Table 1:** Microcirculatory parameters of the regions of interest*

Parameter	Basic values	Postburn values	Day 1 postburn	Day 3 postburn	Day 7 postburn	Day 14 postburn
Nonperfused area, mm^2^			1.28 ± 0.02	0.82 ± 0.03†	0.41 ± 0.06†	0.003 ± 0.003†
Functional vessel density, cm/cm^2^	181.6 ± 4	135.2 ± 5.1†	134.9 ± 8.6†	139.1 ± 7†	131.3 ± 7.3†	144.5 ± 6†
Diameter venules, µm	24.5 ± 1.94	27.74 ± 1.98‡	30.41 ± 2.17†	28.34 ± 2.32‡	28.67 ± 2.67†	29.6 ± 2.11†
Diameter arterioles, µm	21.66 ± 1.62	26.83 ± 1.45†	25.42 ± 1.95‡	25.42 ± 2.44‡	24.03 ± 2.24	24.63 ± 1.78
Red blood cell velocity, mm/s	0.25 ± 0.02	0.37 ± 0.05†	0.3 ± 0.05	0.3 ± 0.05	0.22 ± 0.05	0.23 ± 0.03
Leakage, *I*_*e*_/*I*_*i*_	0.55 ± 0.02	1 ± 0.04†	0.8 ± 0.01†	0.72 ± 0.01†	0.79 ± 0.04†	0.74 ± 0.03†
Rolling leukocytes, *n*/20 s	4.9 ± 0.6	5.3 ± 0.7	7.2 ± 0.8‡	5.2 ± 0.7	4.3 ± 0.6	5.2 ± 0.8
Sticking leukocytes, *n*/100 µm	0.9 ± 0.3	3.2 ± 0.4†	3.7 ± 0.3†	1 ± 0.2	0.7 ± 0.3	0.2 ± 0.1‡

* Data are given as mean ± standard error of mean (see also Fig. 2).

† *P* < .01 versus baseline values.

‡ *P* < .05 versus baseline values.
